# Assessment of Hypoxia in the Stroma of Patient-Derived Pancreatic Tumor Xenografts

**DOI:** 10.3390/cancers6010459

**Published:** 2014-02-26

**Authors:** Ines Lohse, Corey Lourenco, Emin Ibrahimov, Melania Pintilie, Ming-Sound Tsao, David W. Hedley

**Affiliations:** 1 Ontario Cancer Institute and Campbell Family Cancer Research Institute, Princess Margaret Cancer Center, University Health Network, 610 University Ave., Toronto, ON M5G2M9, Canada; E-Mails: ilohse@uhnresearch.ca (I.L.); coreylourenco@gmail.com (C.L.); emin.ibrahimov@utoronto.ca (E.I.); pintilie@uhnresearch.ca (M.P.); Ming.Tsao@uhn.ca (M.-S.T.); 2 Department of Pathology, University Health Network, 200 Elizabeth Street, Toronto, ON M5G2C4, Canada; 3 Department of Laboratory Medicine and Pathobiology, 27 King’s College Circle, University of Toronto, Toronto, ON M5S1A1, Canada; 4 Departments of Medical Biophysics University of Toronto, 610 University Ave., Toronto, ON M5G2M9, Canada; 5 Departments of Medicine, University of Toronto, 610 University Ave., Toronto, ON M5G2M9, Canada; 6 Department of Medical Oncology and Hematology, Princess Margaret Cancer Center, 610 University Ave., Toronto, ON M5G2M9, Canada

**Keywords:** pancreatic cancer, tumor hypoxia, tumor-associated stroma, patient-derived pancreatic xenograft models, pattern recognition software

## Abstract

The unusually dense stroma of pancreatic cancers is thought to play an important role in their biological aggression. The presence of hypoxia is also considered an adverse prognostic factor. Although it is usually assumed that this is the result of effects of hypoxia on the epithelial component, it is possible that hypoxia exerts indirect effects via the tumor stroma. We therefore measured hypoxia in the stroma of a series of primary pancreatic cancer xenografts. Nine patient-derived pancreatic xenografts representing a range of oxygenation levels were labeled by immunohistochemistry for EF5 and analyzed using semi-automated pattern recognition software. Hypoxia in the tumor and stroma was correlated with tumor growth and metastatic potential. The extent of hypoxia varied from 1%–39% between the different models. EF5 labeling in the stroma ranged from 0–20% between models, and was correlated with the level of hypoxia in the tumor cell area, but not microvessel density. Tumor hypoxia correlated with spontaneous metastasis formation with the exception of one hypoxic model that showed disproportionately low levels of hypoxia in the stroma and was non-metastatic. Our results demonstrate that hypoxia exists in the stroma of primary pancreatic cancer xenografts and suggest that stromal hypoxia impacts the metastatic potential.

## 1. Introduction

A characteristic feature of pancreatic cancer is the dense desmoplastic reaction that can account for up to 80% of the total tumor mass [1,2]. It is believed to develop by paracrine and autocrine signaling between tumor cells, tumor-associated fibroblasts, pancreatic stellate cells, inflammatory cells and the extracellular matrix (ECM), and can be referred to as the tumor microenvironment [2,3,4,5]. The interactions between tumor cells and their microenvironment are complex, but the unusually dense stromal reaction seen in pancreatic cancers is thought to play an important role promoting their high lethality [3,4,5,6]. 

Hypoxia probably develops in all solid tumors as the utilization of oxygen exceeds its delivery by an inefficient neovasculature, although the extent of hypoxia is very variable between different tumors of the same histological type and stage [7,8,9]. Hypoxia and its associated metabolic changes are considered to be an additional component of the tumor microenvironment, and it has long been associated with poor patient outcome [10,11,12,13,14,15,16]. Early work focused on the effects of hypoxia on resistance to radiotherapy and to chemotherapy [14,15,16], but recently there has been recognition that hypoxia influences tumor cells to enhance invasion and metastasis. There is also some evidence that hypoxia favors the maintenance of a stem cell-like phenotype [17,18,19]. 

Pancreatic cancers have been thought to be unusually hypoxic based on the poor perfusion of radiological contrast agents, and a limited experience with direct pO_2_ measurements [7]. However, recently we showed that primary pancreatic cancer xenografts show a range in values similar to that reported for other cancer types [10]. 

Although the dense stroma and the presence of hypoxia are each to be considered components of the pancreatic cancer microenvironment, there has been surprisingly little work asking if hypoxia influences the stroma directly, rather than via effects on the cancer cells. For example, the secretion of growth factors or cytokines by mesenchymal cells might be influenced by hypoxia so as to affect paracrine interactions with the cancer cells. To address this question, we undertook an analysis of the distribution of hypoxia in both the epithelial and stromal compartments of a series of primary pancreatic cancer xenografts selected to represent a wide range of hypoxia and *in vivo* growth characteristics.

## 2. Results and Discussion

### 2.1. Primary Patient-Derived Pancreatic Xenograft Models Display Different Levels of Tumor Hypoxia

Primary xenografts were established from pancreatectomy samples that were superfluous to diagnostic needs by implantation into the flank or to the surface of the pancreas of NOD/SCID mice. It has been previously demonstrated that these xenografts closely resemble the patient tumor they were derived from and represent a range of morphology that has been described for patient specimen [10].

**Figure 1:**
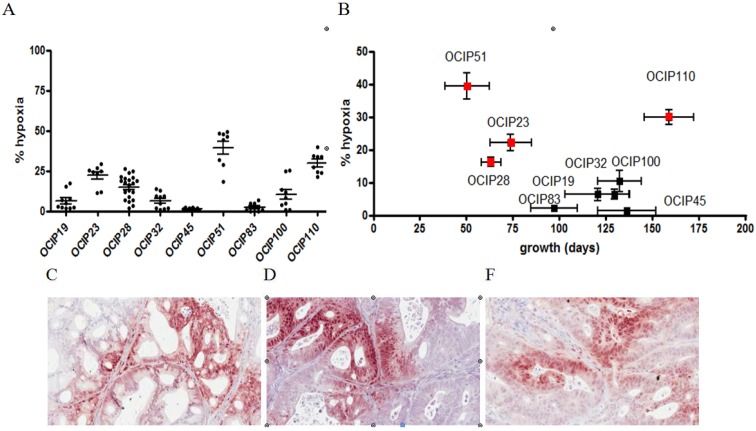
Patient-derived pancreatic xenograft models differ in the magnitude of tumor hypoxia and metastatic potential. (**A**) Percentage of tumor hypoxia as indicated by EF5 staining of the patient-derived xenograft models. Each point represents the hypoxia status in a single tumor. The xenograft models display a wide range of tumor hypoxia that remains similar over several passages *in vivo*; (**B**) EF5 staining vs. tumour growth shows that hypoxia mostly correlates with rapid growth pattern. Tumor growth is defined by the time elapsed between two passages. Metastatic models are labelled in red. Representative sections of (**C**) OCIP19, (**D**) OCIP23 and (**E**) OCIP51 stained for EF5. Tumor hypoxia in all models is found in both the tumor and tumor-associated stroma.

**Figure A1:**
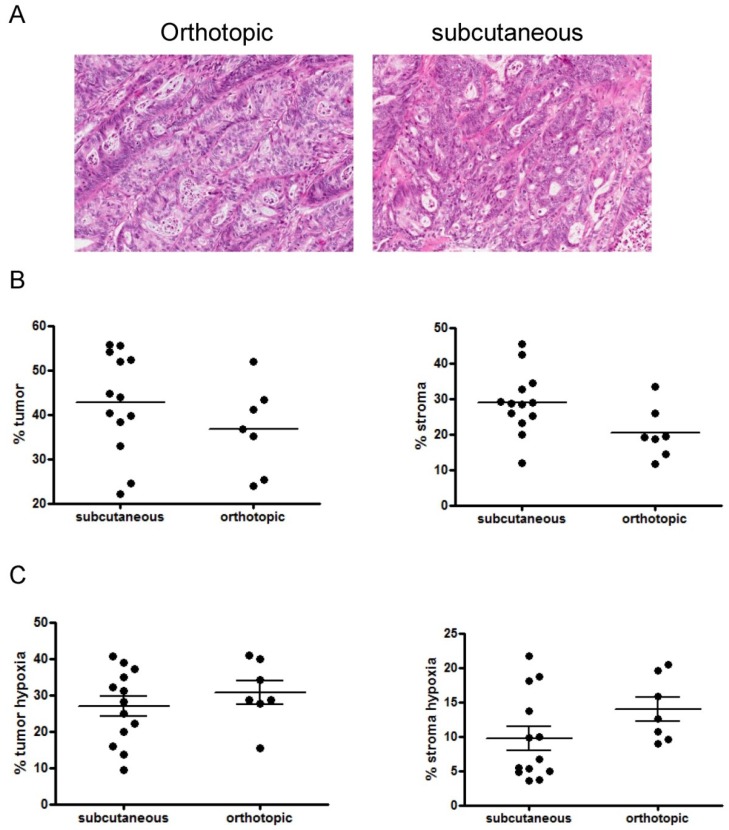
The patient-derived xenografts display similar characteristics at both the subcutaneous and orthotopic implantation site. Xenografts maintain stable (**A**) morphology, (**B**) tumor and stroma content and (**C**) hypoxia levels at both the subcutaneous and orthotopic implantation site as displayed on the example of OCIP23.

The primary patient-derived xenografts used for this study displayed a wide range of tumor hypoxia (1%–39%) that was highly significant (*p* < 0.0001), whereas there was relatively little variance in the levels of hypoxia within replicates from the same model (Figure 1A). The level of tumor hypoxia, however, did not depend on the size of the examined tumor but was similar in tumors of the same model independent of the tumor diameter. All models show stable morphology and hypoxia levels over several passages in NOD/SCID mice. Additionally no differences in tumor morphology and the magnitude of tumor hypoxia were observed between tumors implanted at either implantation site (Appendix Figure A1), which allowed us to use tumors from both implantation sites for the subsequent analysis.

In the models used for this study, high levels of hypoxia generally correlated with rapid tumour growth and high metastatic potential (Figure 1B). As illustrated in Figure 1C–E), positive EF5 staining was seen in the stroma as well as the epithelial compartments, and we therefore developed an automated image analysis routine to quantify this effect. 

### 2.2. Xenograft Models Significantly Differ in Tumor and Stroma Composition

**Figure 2:**
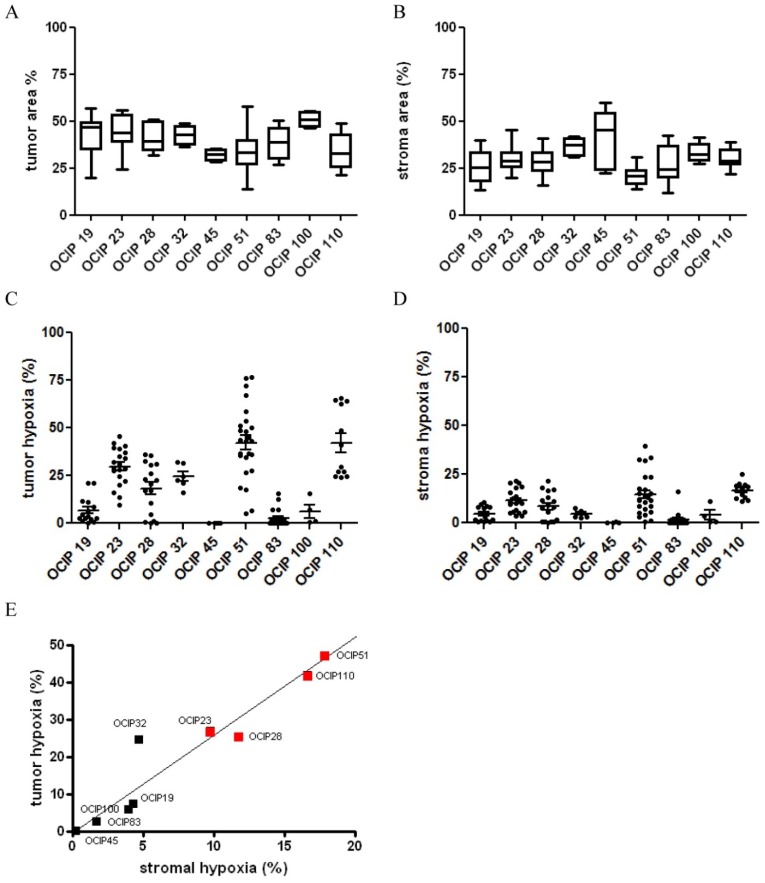
Patient-derived pancreatic xenograft models differ in tumor and stroma content. Classification of EF5 stained sections using the Aperio pattern recognition software. Sections of tumors were examined for (**A**) epithelial and (**B**) stromal content. Analysis of the hypoxic fraction in the (**C**) epithelial and (**D**) stromal compartment of EF5 stained sections of tumors. Stromal hypoxia, although overall displaying a lower magnitude, shows the same pattern of high hypoxic and low hypoxic models that can be established from the analysis of the epithelial hypoxia; (**E**) Tumor hypoxia in the epithelial compartment strongly correlated with the stromal compartment in sections stained for the hypoxia marker EF5 using the Aperio pattern recognition software. Metastatic potential (red square) to liver and peritoneum was observed in models with high tumor and stromal hypoxia.

**Figure A2:**
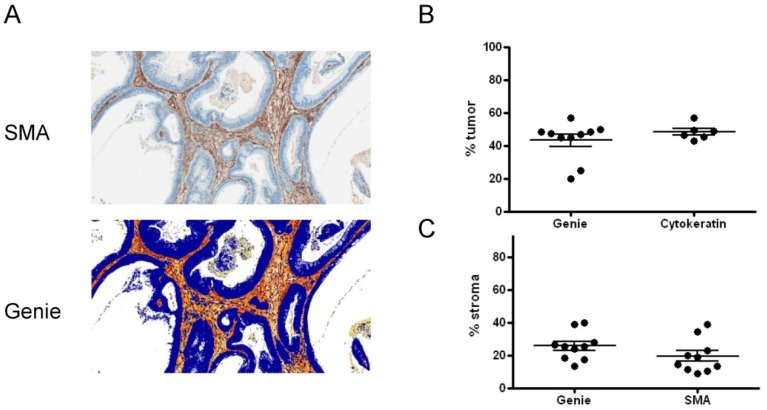
The Genie pattern recognition software can be used to evaluate tumor and stoma content. (**A**) Representative images of α-SMA (top) and EF5 stained sections classified with the stroma classifier (bottom). Classification of the (**B**) epithelial and the (**C**) stromal compartment using the Genie classifiers closely resembles the histological staining for cytokeratin and α-SMA, respectively.

To test the specificity of the Genie classifiers adjacent sections of OCIP19 were stained for EF5, α-SMA and cytokeratin. The EF5 stained sections were examined for tumor and stroma content using the Genie software (Appendix Figure A2A). The tumor cell content was similar when established by either the Genie classifiers or the cytokeratin staining (Appendix Figure A2B). The stroma content however was higher when examined using the Genie software if compared to the α-SMA staining because the Genie classifiers recognize both the cellular as well as the fibrous stroma (Appendix Figure A2C). The individual models displayed an intra-model range of tumor and stroma content (Figure 2A,B). Furthermore, the individual models differed significantly in their tumor (*p* = 0.0006) and stroma (*p* < 0.0001) content (Figure 2A,B). The highest stroma content was observed in the low hypoxic models OCIP45 and 32. However, no correlation between the extent of tumor-associated stroma and tumor hypoxia was observed in these models. Similar, the extent of stromal content did not correlate with tumor growth rate or metastatic potential.

### 2.3. Analysis of Epithelial *versus* Stroma Hypoxia

To investigate the hypoxic fraction within the tumor and stroma separately, the PPC algorithm was applied to the tumor and stroma areas established using the Genie software, and the results are summarized in Figure 2. Similar to the distribution of hypoxia in the whole sections (Figure 1A), there was considerable heterogeneity in the levels of hypoxia between the individual models, whereas within models the results were relatively similar. The levels of hypoxia in the tumor associated stroma were generally lower when compared to the tumor compartment (Figure 2C,D). There was a strong correlation between the stroma and epithelial compartments between the individual models (R = 0.918), with the exception of OCIP32 where the level of hypoxia in the stroma appeared disproportionately low compared to the epithelium (Figure 2E). While all other models displaying high levels of tumor hypoxia also showed high metastatic potential, no metastases were observed in OCIP32 bearing animals. Metastatic potential was assessed through the presences of metastases on the surface of liver and spleen of mice bearing orthotopically implanted tumors.

### 2.4. Relationship between Hypoxia and Vessel Density

**Figure 3:**
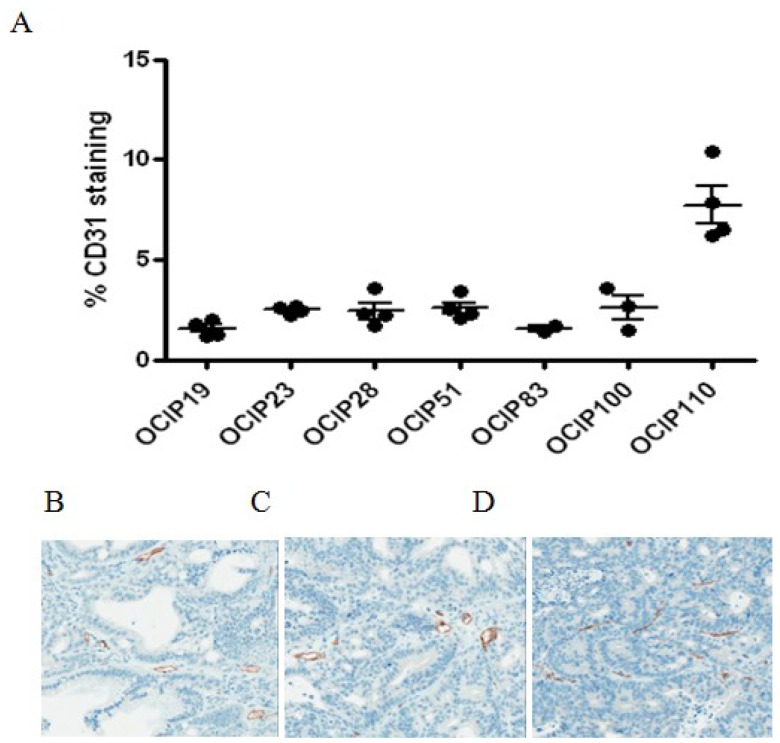
Patient-derived pancreatic xenograft models differ in microvessel density. (**A**) Percentage of CD31 of the patient-derived xenograft models. Each point represents a single tumor. In these models, staining for CD31 indicating vessel density shows little difference between the tested models and shows no correlation with tumor hypoxia. Representative sections of (**B**) OCIP19, (**C**) OCIP23 and (**D**) OCIP51 stained for CD31.

Blood vessels were readily identified by CD31 staining in the stroma of all models (Figure 3). Automated analysis of microvessel density yielded similar values within and between the individual models, with the exception of OCIP110, where the levels were much higher. We did however observe differences in vessel morphology between the xenograft models (Figure 3B). While models displaying low and medium levels of hypoxia display open vascular lumen, highly hypoxic models like OCIP51 feature vessels with small or no vascular lumen. No statistically significant associations were seen between vessel density and the extent of hypoxia in the epithelial or stroma compartments of the tumors. 

### 2.5. Discussion

Desmoplasia is a prominent and well described feature of pancreatic cancer that distinctively sets these tumors apart from other adenocarcinomas. Although cases have been described featuring large areas of tumor-associated stroma comprising up to 80% of the total tumor mass, the stromal content of the majority of tumors is significantly lower [1,2,4].

Nevertheless the extent of tumor-associated stroma in pancreatic tumors suggests an important role in providing a permissive environment that not only facilitates tumor cell survival but may also promote tumor growth and early systemic dissemination observed in pancreatic cancer patients [4,5,9].

Pancreatic carcinomas have been reported to be some of the most hypoxic of human tumors, although the data supporting this view is mostly limited to early Eppendorf probe measurements and HIF1α staining [7,20,21]. Our studies with pancreatic cancer xenografts and initial studies in patients suggest that like other solid tumors, they can display a wide range of oxygenation levels [10]. The presence of HIF1α positive cells within the stromal compartment has been reported by a number of different studies [20,21], but the correlation between hypoxia in the tumor and stroma compartments as well as its relevance for markers of tumor aggressiveness such as growth rate and metastasis has not been evaluated to date and is the main focus of this study.

We examined a set of nine patient-derived pancreatic xenografts featuring a wide range of tumor hypoxia as established using the hypoxia marker EF5. In contrast to the use of HIF1α staining which may lead to artifacts attributed to the surgical resection of the tumor or delayed fixation, the magnitude of EF5 within the tumor remains stable once the molecule has been metabolized.

Although the use of chimeric models can be disadvantageous because of incompatibilities between the mouse stroma and the human tumor cells, the patient-derived xenografts used in this study provide us with the unique opportunity to investigate the influence of stromal hypoxia on the tumor phenotype in a well-defined model displaying a wide range of hypoxia levels and growth characteristics. This allowed us to not only examine the magnitude of tumor hypoxia but also to correlate the data with clinical markers of tumor aggressiveness such as tumor growth rate and metastatic potential.

Tumor hypoxia has been shown to activate a multitude of signaling pathways that result in rapid tumor growth and increased tumor cell motility [17,18,19]. Indeed, we previously reported that high levels of tumor hypoxia in patient-derived pancreatic xenografts correlate with rapid growth and high metastatic potential [10]. Additionally, Erkan *et al*. have suggested that the high stroma content of pancreatic tumors results in increased tumor hypoxia as a result of abnormal extracellular matrix deposition [4]. In our data set, however, we did not observe a correlation between high levels of tumor hypoxia and stromal content even though the range of stromal content is similar to what has been observed in patients. Indeed, the xenograft model displaying the highest stromal fraction was also the model with the lowest level of hypoxia in both the tumor and stroma compartment. Although this seems to contradict the observation made by Erkan *et al*. [4], we hypothesize that some of the difference are due to the model systems used in either study. 

The examination of the magnitude of hypoxia in the tumor and stroma regions separately showed a range of hypoxia that was generally similar to the range in whole tumor hypoxia. With the exception of OCIP32, tumor hypoxia strongly correlates with tumor hypoxia, although the magnitude of hypoxia was generally lower in stromal regions. Even though most blood vessels are located within the stroma, highly hypoxic models like OCIP51 feature large areas of stromal hypoxia. Still, we found no correlation between the number of microvessels and tumor hypoxia, although we observed differences in vessel morphology in hypoxic models resulting in a reduction of vascular lumen. 

In the patient-derived pancreatic xenografts described here, high levels of tumor hypoxia generally correlate with rapid tumor growth and high metastatic potential. OCIP110 however despite displaying high levels of hypoxia in both the tumor and stroma compartment and metastases to the liver, features a slow growth rate suggesting that stromal hypoxia may not influence tumor growth rate in this model system. OCIP32 on the other hand, while showing levels of tumor hypoxia similar to the metastatic models showed a disproportionately low level of hypoxia in the stroma, and features a slow growth pattern and low metastatic potential. Although hypoxia has been shown to increase tumor cell motility, the effect of stromal hypoxia on the metastatic cascade has yet to be elucidated. Based on the results present here, we hypothesize that stromal hypoxia may play a role in facilitating tumor metastasis. 

## 3. Experimental

### 3.1. Primary Patient-Derived Xenografts

Animal experiments were carried out using protocols (AUP791, 11/11/05) approved by University Health Network (UHN) Animal Care Committee under the guidelines of the Canadian Council on Animal Care. Subcutaneous and orthotopic tumors of nine primary xenografts, designated as Ontario Cancer Institute Pancreas (OCIP) 19, 23, 28, 32, 45, 51, 83, 100 and 110, were established from pancreatectomy samples using a protocol approved by the University Health Network Research Ethics Board. Written informed consent was obtained from all participating patients and the consent procedure was reviewed by the University Health Network Research Ethics Board.

Tumors were established in 4- to 5-week non-obese diabetic severe immune deficient mice (NOD/SCID) by implantation of tumor fragments into the flank or to the surface of the pancreas under general anesthesia. Tumors were grown at either implantation site to a size of approximately 1cm in diameter. Metastatic potential to liver and peritoneum was evaluated in animals bearing orthotopic tumors.

### 3.2. Immunohistochemical Analysis

To determine the level of tumor hypoxia, mice were injected intraperitoneally (ip) with the 2-nitroimidazole hypoxia marker EF5, 30 mg/kg, 3 h prior to sacrifice [8,22,23]. Tumors were excised, fixed and paraffin embedded. Paraffin tissue sections were cut, dried and dewaxed. Endogenous peroxidase was blocked in 3% hydrogen peroxide for 10 min. Microwave antigen retrieval was carried out under pressure at 120C for 10 min in 10 mM Citrate buffer, pH 6.0 using a T/T Mega microwave oven. Endogenous biotin was blocked in Vector’s biotin blocking kit, and then slides were labeled with primary antibodies to α-Smooth Muscle Actin (α-SΜΑ, (DAKO, Glostrup, Denmark, clone 1A4, 1:400), CD31 (Santa Cruz, Dallas, TX, USA, sc-1506, 1:1000) or cytokeratin (DAKO, Glostrup, Denmark, AE1/AE3, 1:200) overnight. Biotinylated anti-mouse IgG incubations were carried out followed by streptavidin biotin detection system (Signet Pathology System, Deham, MA, USA) for 30 min each. For EF5 staining, slides were incubated in biotinylated EF5 antibody (obtained from Dr. Cameron Koch, University of Pennsylvania, Philadelphia, PA, USA) at 1:250 overnight, followed by streptavidin biotin detection system (Signet Pathology System) for 30 min each. Immunoreactivities were revealed by incubation in Nova Red substrate (Vector Lab, Burlingame, CA, USA) for 5 min and counterstained in Mayer’s haematoxylin.

### 3.3. Image Analysis

Sections were scanned at 20× resolution using an Aperio Scanscope XT scanner (Aperio Technologies, Vista, CA, USA). Images were analyzed using the Aperio ImageScope software ver. 11.1.2.752, positive pixel count algorithm (PPC) [24]. No image processing was carried out prior to the analysis. The Genie pattern recognition software is an extension of the Aperio ImageScope software and can be trained to recognize and classify different histological features within the tumor [25,26].

Training is based on representative regions of tumor, stroma, ductal space and tumor necrosis. In order to compensate for morphological difference between the xenograft models, individual classifiers were trained for every xenograft model. The training was based on different slides until the classification of the different morphological features until the visual examination of test sections appeared optimal and no further improvement could be achieved by additional training cycles. Once the classifiers are trained, each classification layer is analyzed separately using the PPC algorithm to quantify hypoxia.

### 3.4. Statistical Analysis

A linear regression model was employed to investigate the differences between the level of hypoxia for the different models, and between tumor and stromal compartments as well as for testing the differences between the tumor and stromal content. Pearson correlation coefficient was calculated between the hypoxia in the tumor and stroma.

## 4. Conclusions

Similar to the clinical situation, primary xenografts derived from pancreatic cancer patients lay down a fibrovascular stroma that makes up 25% to 50% of the total volume. High levels of hypoxia develop in some models, whereas others show very low levels. Unexpectedly, high levels of hypoxia also occur in the stroma of some models independently of microvessel density. Although there is an extensive literature of describing the effect of hypoxia on cancer cell biology, surprisingly little work has been done to describe the potential effects of hypoxia on the tumor stroma, and it’s potential to effect tumor growth and metastasis. The results presented here suggest that further work should be done in this area.
